# Supermodal Decomposition of the Linear Swing Equation for Multilayer Networks

**DOI:** 10.1109/access.2022.3188392

**Published:** 2022-07-04

**Authors:** KSHITIJ BHATTA, AMIRHOSSEIN NAZERIAN, FRANCESCO SORRENTINO

**Affiliations:** 1 Department of Mechanical and Aerospace Engineering, Univeristy of Virginia, Charlottesvile, VA 22903, USA; 2 Mechanical Engineering Department, University of New Mexico, Albuquerque, NM 87131, USA

**Keywords:** Modal decomposition, multi-layer networks, quadratic eigenvalue problem, swing equation

## Abstract

We study the swing equation in the case of a multilayer network in which generators and motors are modeled differently; namely, the model for each generator is given by second order dynamics and the model for each motor is given by first order dynamics. We also remove the commonly used assumption of equal damping coefficients in the second order dynamics. Under these general conditions, we are able to obtain a decomposition of the linear swing equation into independent modes describing the propagation of small perturbations. In the process, we identify symmetries affecting the structure and dynamics of the multilayer network and derive an essential model based on a ‘quotient network.’ We then compare the dynamics of the full network and that of the quotient network and obtain a modal decomposition of the error dynamics. We also provide a method to quantify the steady-state error and the maximum overshoot error. Two case studies are presented to illustrate application of our method.

## I. INTRODUCTION

Modeling and analysis of power grid dynamics have been the subject of many papers [1], [9], [12], [13], [15], [16], [20], [21], [26], [29], [34], [35], [38], [39], [41], [48]. A fundamental tool to describe the dynamics of power grids is the swing equation. In the presence of small disturbances, this equation can be approximated by the linear swing equation, which can be decomposed into independent modes [2], [5], [19], [24], [37], [46]. However, most papers in the literature introduce two unrealistic assumptions in order to derive a modal decomposition: 1- the same model is used for both generators and motors, while they are intrinsically different and 2- all the individual systems are characterized by the same effective damping. An exception to 2 is provided by Tyloo *et al*. [46] who relax the constant inertia to damping ratio assumption and show that their derivation is still valid with heterogeneous dynamical parameters. Here we remove both assumptions 1 and 2. We introduce intrinsically different mathematical models for generators and motors, where motors have typically negligible inertia compared to generators and we allow for the damping terms to be arbitrarily chosen. After removing both assumptions 1 and 2 we show that a modal decomposition of the linear swing equation can still be obtained.

While performing this analysis we also look for the effects of symmetries in the network topology. Symmetries play a significant role in the study of networked systems. References [3], [6], [11], [14], [17], [31], [33], [36], [43]–[45], [49] have proposed tools based on graph theory and group theory to analyze the dynamics of complex networks with symmetries. A recent paper [10] has proposed indices for the characterization of symmetries in complex networks. Reference [7] has discussed how the analysis of symmetries may be used to explore associations in image features to infer similarities/dissimilarities among features.

The presence of symmetries in power grid networks has been documented in [14], [45]. Reference [22] has analyzed how network symmetries may affect the synchronization modes of power grid networks, while [18] has studied structure and dynamics preserving reduction methods for power grids using a graph theoretical approach. Reference [5] has analyzed the effect of symmetries in networks using the linear swing equation and developed techniques for easy calculation of maximum flow and error using a modal decomposition. Reference [27] has found that certain heterogeneous parameters enhance the stability of power grid networks, which provides a motivation for us to remove the assumption of homogeneous parameters. Many real systems do not possess exact symmetries but approximate symmetries. Recent work has investigated the presence of such approximate symmetries in both natural and engineering systems and found that approximate symmetries are widespread in these systems, see e.g., [28], [30]. Reference [42] has shown a connection between the stability of symmetric states and approximately symmetric states.

Different from large part of the literature, in this paper we model powergrids as multilayer networks, where one layer is entirely made up of generator nodes and the other layer of motor nodes (‘loads’). We first obtain a simplified description of the multilayer network dynamics in terms of its quotient network. This description is useful as it provides an essential model for the dynamics in which redundancies due to symmetry are ‘removed’ [44], under the assumption that symmetric nodes produce/absorb the same amount of power. In what follows, we withdraw this assumption and quantify the error/deviation dynamics between the quotient network model and the full network model. Further, we develop a method to compute the maximum overshoot of the aforementioned error dynamics.

The are several novel contributions of this paper, which we briefly review next. They are: (i) a study of the linear swing equation applied to a multilayer network in which loads and generators are modeled differently, (ii) a dynamical reduction of the dynamics in the case of heterogeneous damping coefficient based on the solution of the quadratic eigenvalue problem, (iii) an expansion of the solution in terms of first order supermodes and second-order supermodes, and (iv) a characterization of the error dynamics of the quotient network when compared to the full-network in terms of supermodes.

The rest of the paper is organized as follows. Section II covers the modeling of a powergrid as a multilayer network using the linear swing equation and block diagonalization of the network equation into its quotient block and transverse block using the IRR transformation. Section III describes the block-diagonalization of the error dynamics. Section IV deals with the maximum error computation using the concept of a ‘supermode’ and Section V provides validation using one example network and one real-life power grid network. Finally, conclusions are given in Section VI.

## II. NETWORK MODEL

Several alternative models of the swing equation have been proposed [25], [32]. For this paper, we specialized Bergen and Hill’s Structure Preserving Model [4], [32] to multilayer networks. The power grid is formed of a collection of rotating machines (‘nodes’) connected by transmission lines (‘edges’). Nodes can be of either one of two types: generator nodes which produce power, and motor nodes (or loads) which consume power and have typically a lower inertia than generator nodes.

We thus consider a multilayer network with two layers: the generator layer denoted G with *n*_*G*_ nodes and the load layer denoted L with *n*_*L*_ nodes. The nodes in G are modeled as second order oscillators, with dynamics described by the following equation,

(1) $${\ddot{\theta }}_{i}+\gamma_{i}{\dot{\theta }}_{i}=q_{i}^{G}-\sum_{j=1,j\neq i}^{n_{G}}{\tilde{A}}_{ij}^{G}\text{ sin}\left(\theta_{i}-\theta_{j}\right)\;-\sum_{j=1}^{n_{L}}{\tilde{A}}_{ij}^{GL}\text{ sin}\left(\theta_{i}-\phi_{j}\right),\;i=1,2,\;\ldots \;n_{G}$$

where *θ*_*i*_(*t*) is the nodal displacement of generator node *i*, *γ*_*i*_ > 0 is the damping coefficient of generator *i* and $q_{i}^{G}>0$ is the power produced at generator node *i*. The nodes in L are modeled as first order oscillators which obey the following equation,

(2) $${\dot{\phi }}_{j}=q_{j}^{L}-\sum_{i=1,i\neq j}^{n_{L}}{\tilde{A}}_{ji}^{L}\text{ sin}\left(\phi_{j}-\phi_{i}\right)-\sum_{i=1}^{n_{G}}{\tilde{A}}_{ji}^{LG}\text{ sin}\left(\phi_{j}-\theta_{i}\right),\;j=1,2,\;\ldots \;n_{L},$$

where *ϕ*_*j*_(*t*) is the nodal displacement of motor node *j*, and $q_{j}^{L}<0$ is the power consumed at load *j*.

*Definition 1: The network is said to be balanced if* $\sum_{i=1}^{n_{G}}q_{i}^{G}+\sum_{j=1}^{n_{L}}q_{j}^{L}=0$.

We assume the network is balanced throughout the paper unless specified otherwise. The multilayer network can be represented using the ‘Supra-Adjacency matrix’ which is an *n* × *n* matrix, *n* = *n*_*G*_ + *n*_*L*_,

$$\tilde{A}=\left[\begin{array}{ll} {\tilde{A}}^{G} & {\tilde{A}}^{GL}\\ {\tilde{A}}^{LG} & {\tilde{A}}^{L} \end{array}\right],$$

where the *n*_*G*_ × *n*_*G*_-dimensional symmetric matrix ${\tilde{A}}^{G}=\left\{{\tilde{A}}_{ij}^{G}\right\}$ (the *n*_*L*_ × *n*_*L*_-dimensional symmetric matrix ${\tilde{A}}^{L}=\left\{{\tilde{A}}_{ij}^{L}\right\}$) describes the intra-layer connectivity of layer G(L.) Namely, ${\tilde{A}}_{ij}^{G}={\tilde{A}}_{ji}^{G}>0$ if generator nodes *i* and *j* are connected and ${\tilde{A}}_{ij}^{L}={\tilde{A}}_{ji}^{L}=0$ otherwise. Analogously, ${\tilde{A}}_{ij}^{L}={\tilde{A}}_{ji}^{L}>0$ if motor nodes *i* and *j* are connected and ${\tilde{A}}_{ij}^{L}={\tilde{A}}_{ji}^{L}=0$ otherwise. The interlayer connectivity is given by the *n*_*G*_ × *n*_*L*_-dimensional matrix ${\tilde{A}}^{GL}=\left\{{\tilde{A}}_{ij}^{GL}\right\}$ and by the the *n*_*L*_ × *n*_*G*_-dimensional matrix ${\tilde{A}}^{LG}=\left\{{\tilde{A}}_{ij}^{LG}\right\}$, ${\tilde{A}}^{LG}={\tilde{A}}^{GL^{T}}$. If generator node *i* and motor node *j* are connected (not connected), then ${\tilde{A}}_{ij}^{GL}={\tilde{A}}_{ji}^{LG}>0\left({\tilde{A}}_{ij}^{GL}={\tilde{A}}_{ji}^{LG}=0.\right)$

Like in the case of single-layered networks, multilayer networks may be affected by the presence of symmetries [14]. In general, a symmetry is a permutation of the network nodes that results in a network that is isomorphic to the original one. The symmetry group S is the set of all symmetries with the operation composition. The set of all symmetries in the group will only permute certain subsets of nodes (the *orbits* or *clusters*) among each other. The nodes in each subset are mapped into each other by application of one or more symmetries in S; however, there is no symmetry in S that will map into each other nodes in different subsets. We refer to such subsets of nodes as ‘clusters’ or ‘orbits’ of the symmetry group [5].

For the case of multilayer networks, with layers composed of nodes of different types, the above definition of symmetry needs to be specialized to account for the fact that symmetries may only move nodes (may not move nodes) from the same layer (from different layers.)

We first introduce the group of symmetries for each layer: $s_{G}\in S_{G}$ for layer G which satisfies,

(3) $$s_{G}{\tilde{A}}^{G}={\tilde{A}}^{G}s_{G}$$

and $s_{L}\in S_{L}$ for layer L, which satisfies,

(4) $$s_{L}{\tilde{A}}^{L}={\tilde{A}}^{L}s_{L}.$$

*Definition 2: A symmetry* P∈S *for the set of* (1) *and* (2) *is defined as a block diagonal permutation matrix of the form*,

$$P=\left[\begin{array}{cc} s_{G} & 0\\ 0 & s_{L} \end{array}\right]$$

*with* $s_{G}\in S^{G}$, $s_{L}\in S^{L}$, *and such that* $P\tilde{A}=\tilde{A}P$ *and s*_*G*_***γ*** = ***γ***, *where the n*_*G*_-*dimensional vector* $\gamma =\left[\gamma_{1},\gamma_{2},\;\ldots \;,\gamma_{n_{G}}\right]$.

In addition to the equalities (3) and (4), the equation $P\tilde{A}=\tilde{A}P$ also requires satisfaction of the following two conjugacy relationships [14],

(5a) $$s_{L}{\tilde{A}}^{LG}={\tilde{A}}^{GL}s_{G}$$

(5b) $$s_{G}{\tilde{A}}^{GL}={\tilde{A}}^{LG}s_{L}.$$

By using (5a) and (5b), we can define the following two subgroups of $S^{G}$ and $S^{L}$ [14],

$$H^{G}=\left\{s_{G}\in S^{G}|s_{G}{\tilde{A}}^{GL}={\tilde{A}}^{GL}s_{L}\text{ and, }s_{L}{\tilde{A}}^{LG}={\tilde{A}}^{LG}s_{G}\text{ for }s_{L}\in S^{L}\right\}$$

$$H^{L}=\left\{s_{L}\in S^{L}|s_{L}{\tilde{A}}^{LG}={\tilde{A}}^{LG}s_{G}\text{ and, }s_{G}{\tilde{A}}^{GL}={\tilde{A}}^{GL}s_{L}\text{ for }s_{G}\in S^{G}\right\}$$

Following [14] we can prove that the two sets $H^{G}$ and $H^{L}$ are subgroups of $S^{G}$ and $S^{L}$, respectively.

The symmetry group S partitions the set of nodes of the generator layer into a set of *l*_*G*_ clusters, $C_{k}^{G}$, *k* = 1, …, *l*_*G*_ and the set of nodes of the load layer into a set of *l*_*L*_ clusters, $C_{k}^{L}$, *k* = 1, …, *l*_*L*_. For each layer, each cluster is formed of nodes that are mapped into each other by application of all the symmetries in S; however there is no symmetry in S that maps into each other nodes in different clusters. We call *l* = *l*_*G*_ + *l*_*L*_ the total number of clusters. In what follows we call i* the cluster to which generator *i* belongs, and j* the cluster to which motor *j* belongs.

*Lemma 1: A flow invariant synchronous solution* $\theta_{i}(t)=\theta_{i*}(t)$, $\phi_{j}(t)=\phi_{j*}(t)$ *is induced by the automorphism group* S.

*Proof:* Assume $\theta_{i}(0)=\theta_{i*}(0)$ and ${\dot{\theta }}_{i}(0)={\dot{\theta }}_{i*}(0)$ for all *i*’s in cluster i* and for all clusters i* = 1, …, *l*_*G*_ and $\phi_{j}(0)=\phi_{j*}(0)$ for all *j*’s in cluster j* and for all clusters j* = 1, …, *l*_*L*_. It follows that ${\ddot{\theta }}_{i}(0)$ is the same for all *i*’s in cluster i* and ${\dot{\phi }}_{j}(0)$ is the same for all *j*’s in cluster j*. □

Next we assume convergence of the flow invariant solution on the fixed points, $\theta_{i*}^{ss}$ for all *i*’s in cluster i*, and $\phi_{j*}^{ss}$ for all *j*’s in cluster j*. The linearized swing equation, which models the propagation of small disturbances (e.g., affecting the initial condition or affecting the power supplied/demanded at different nodes) [46], is obtained by linearizing (1) and (2) about the stable fixed point $\theta_{i*}^{ss}$, *i* = 1, … *n*_*G*_, $\phi_{j*}^{ss}$, *j* = 1, …, *n*_*L*_,

(6) $${\ddot{\vartheta }}_{i}+\gamma_{i}{\dot{\vartheta }}_{i}=p_{i}^{G}+\sum_{j=1}^{n_{G}}L_{ij}^{G}\vartheta_{j}+\sum_{j=1}^{n_{L}}{\hat{A}}_{ij}^{GL}\varphi_{j}-d_{i}^{GL}\vartheta_{i},\;i=1,2,\;\ldots \;n_{G}$$

(7) $${\dot{\varphi }}_{j}=p_{j}^{L}+\overset{n_{L}}{\underset{i=1}{\sum }}L_{ji}^{L}\varphi_{i}+\overset{n_{G}}{\underset{i=1}{\sum }}{\hat{A}}_{ji}^{LG}\vartheta_{i}-d_{j}^{LG}\varphi_{j},\;j=1,2,\;\ldots \;n_{L}$$

where ${\hat{A}}_{ij}^{G}={\tilde{A}}_{ij}^{G}\text{ cos}\left(\theta_{j*}^{ss}-\theta_{i*}^{ss}\right)$, ${\hat{A}}_{ij}^{GL}={\tilde{A}}_{ij}^{GL}\text{ cos}\left(\theta_{j*}^{ss}-\phi_{i*}^{ss}\right)$, ${\hat{A}}_{ji}^{L}={\tilde{A}}_{ji}^{L}\text{ cos}\left(\phi_{j*}^{ss}-\phi_{i*}^{ss}\right)$, ${\hat{A}}_{ji}^{LG}={\tilde{A}}_{ji}^{LG}\text{ cos}\left(\theta_{j*}^{ss}-\phi_{i*}^{ss}\right)$. The Laplacian matrices $L^{G}=\left\{L_{ij}^{G}\right\}$ with entries $L_{ij}^{G}={\hat{A}}_{ij}^{G}-\delta_{ij}\sum_{j}{\hat{A}}_{ij}^{G}$ and $L^{L}=\left\{L_{ji}^{L}\right\}$ with entries $L_{ji}^{L}={\hat{A}}_{ji}^{L}-\delta_{ij}\sum_{i}{\hat{A}}_{ji}^{G}$, where *δ*_*ij*_ is the Kronecker delta. Also, $d_{i}^{GL}=\sum_{j=1}^{n_{L}}{\hat{A}}_{ij}^{GL}$ represents the total connectivity between generator *i* and the L layer and $d_{j}^{LG}=\sum_{i=1}^{n_{G}}{\hat{A}}_{ji}^{LG}$ represents the total connectivity between load *j* and the G layer. Each term $p_{i}^{G}$ and $p_{i}^{L}$ on the right hand side of (6) and (7) represents a small power deviation.

*Definition 3: A symmetry for the set of* (6) *and* (7) *is defined as a block diagonal permutation matrix of the form*,

$$P=\left[\begin{array}{cc} s_{G} & 0\\ 0 & s_{L} \end{array}\right],$$

*with* $s_{G}\in H^{G}$, $s_{L}\in H^{L}$, *and such that* $P\hat{A}=\hat{A}P$, *where the matrix*,

$$\hat{A}=\left[\begin{array}{ll} {\hat{A}}^{G} & {\hat{A}}^{GL}\\ {\hat{A}}^{LG} & {\hat{A}}^{L} \end{array}\right],$$

*and s*_*G*_***γ*** = ***γ***.

*Lemma 2: The set of* Eqs. (1) *and* (2) *have the same set of symmetries as* Eqs. (6) *and* (7).

*Proof:* We need to prove that (a) a matrix P∈S that commutes with $\tilde{A}$, commutes also with $\hat{A}$ and (b) viceversa. We first prove (a). The matrix *P* permutes with one another nodes in layer G and permutes with one another nodes in layer L. For all *v*, *w* = 1, …, *n*, ${\tilde{A}}_{vw}={\tilde{A}}_{v^{\prime }w^{\prime }}$, where *v*′(*w*′) is the node that *v*(*w*) is mapped to by *P*. Now as *v* is mapped to *v*′, then v* = *v*′*; and as *w* is mapped to *w*′, then w* = *w*′*. From this it trivially follows that: (i) if *v*, w∈G, then $\text{cos}\left(\theta_{v*}^{ss}-\theta_{w*}^{ss}\right)=\text{cos}\left(\theta_{v^{\prime }*}^{ss}-\theta_{w^{\prime }*}^{ss}\right)$, (ii) if *v*, w∈L, then $\text{cos}\left(\phi_{v*}^{ss}-\phi_{w*}^{ss}\right)=\text{cos}\left(\phi_{v^{\prime }*}^{ss}-\phi_{w^{\prime }*}^{ss}\right)$ and (iii) if v∈L and w∈G, then $\text{cos}\left(\theta_{v*}^{ss}-\phi_{w*}^{ss}\right)=\text{cos}\left(\theta_{v^{\prime }*}^{ss}-\phi_{w^{\prime }*}^{ss}\right)$. Thus for all *v*, *w* = 1, …, *n*, ${\hat{A}}_{vw}={\hat{A}}_{v^{\prime }w^{\prime }}$, where *v*′(*w*′) is the node that *v*(*w*) is mapped to by *P*. The proof for (b) is analogous. We start from the observation that ${\hat{A}}_{vw}={\hat{A}}_{v^{\prime }w^{\prime }}$ where *v*′(*w*′) is the node *v*(*w*) is mapped to by *P*, and then by using properties (i), (ii), and (iii) we can prove that for all *v* and *w*, ${\tilde{A}}_{vw}={\tilde{A}}_{v^{\prime }w^{\prime }}$. □

We now consider the vectors $\vartheta =\left[\vartheta_{1},\vartheta_{2},\;\ldots \;\vartheta_{n_{G}}\right]$, $\varphi =\left[\varphi_{1},\varphi_{2},\;\ldots \;\varphi_{n_{L}}\right]$, ${\text{p}}^{\text{G}}=\left[p_{1}^{G},p_{2}^{G},\;\ldots \;,p_{n_{G}}^{G}\right]$, ${\text{p}}^{\text{L}}=\left[p_{1}^{L},p_{2}^{L},\;\ldots \;,p_{n_{L}}^{L}\right]$ to rewrite (6) and (7) as,

(8) $$\ddot{\vartheta }+\Gamma \dot{\vartheta }={\text{p}}^{G}+L^{G}\vartheta +{\hat{A}}^{GL}\varphi -D^{GL}\vartheta ,$$

(9) $$\dot{\varphi }={\text{p}}^{L}+L^{L}\varphi +{\hat{A}}^{LG}\vartheta -D^{LG}\varphi ,$$

where the diagonal matrix Г = {Г_*ij*_} with diagonal entries Г_*ii*_ = *γ*_*i*_ is the damping matrix for the generators, *D*^*GL*^ is the degree matrix of the generators with respect to the loads and *D*^*LG*^ is the degree matrix of the loads with respect to the generators. *D*^*GL*^ is defined as the *n*_*G*_ × *n*_*G*_ diagonal matrix where $D_{ij}^{GL}=\sum_{k=1}^{n_{L}}{\hat{A}}_{ik}^{GL}$, if *i* = *j* and 0 otherwise. *D*^*LG*^ is defined similarly.

We introduce the vector **X**^*T*^ = [***ϑ***^*T*^, ***ϕ***^*T*^] and rewrite (8) and (9) in the compact form,

(10) $$M\ddot{\text{X}}+C\dot{\text{X}}+K\text{X}=\text{f}$$

where $K=\left[\begin{array}{cc} L^{G}-D^{GL} & {\hat{A}}^{GL}\\ {\hat{A}}^{LG} & L^{L}-D^{LG} \end{array}\right]$, $\text{f}=\left[\begin{array}{l} {\text{p}}^{G}\\ {\text{p}}^{L} \end{array}\right]$, $M=\left[\begin{array}{ll} I_{n_{G}\times n_{G}} & 0_{n_{G}\times n_{L}}\\ 0_{n_{L}\times n_{G}} & 0_{n_{L}\times n_{L}} \end{array}\right]$ and $C=\left[\begin{array}{cc} \Gamma & 0_{n_{G}\times n_{L}}\\ 0_{n_{L}\times n_{G}} & I_{n_{L}\times n_{L}} \end{array}\right]$.

From knowledge of the group of symmetries S, we can compute the irreducible representations (IRRs) of the symmetry group of the network. This defines a transformation *T* into the so-called IRR coordinate system (see Ref. [36]). The transformation matrix *T* is orthogonal. Each one of the rows of the matrix *T* is associated with a specific layer. If a row of the matrix *T* is associated with layer *k*, all the *i* entries of that row are zero for *i* not in layer *S*_*k*_.

Therefore, the matrix *T* can be cast in the following block diagonal form,

(11) $$T=\underset{k=1,2,\;\ldots \;}{⊕}T^{k},$$

where each block *T*^*k*^ is an *n*_*k*_-dimensional square matrix associated with layer *k* and the symbol ⊕ indicates direct sum of matrices. We will represent these blocks as *T*^*G*^ for the generator layer and *T*^*L*^ for the load layer giving a matrix *T* of the form:

$$T=\left[\begin{array}{cc} T^{G} & 0\\ 0 & T^{L} \end{array}\right].$$

Premultiplying (8) and (9) by *T*, we obtain,

(12) $$\ddot{\xi }(t)=-\Gamma \dot{\xi }(t)+V^{G}\xi (t)+{\overset{*}{A}}^{GL}\kappa (t)-D^{GL}\xi (t)+{\text{r}}^{\text{G}},$$

and

(13) $$\dot{\kappa }\left(t\right)=V^{L}\kappa \left(t\right)+{\overset{*}{A}}^{LG}\xi \left(t\right)-D^{LG}\kappa \left(t\right)+{\text{r}}^{\text{L}}.$$

where ***ξ*** = *T*^*G*^***θ***, ***κ*** = *T*^*L*^***ϕ***, **r**^*G*^ = *T*^*G*^**p**^**G**^, **r**^***L***^ = *T*^*L*^**p**^**L**^, *V*^*G*^ = *T*^*G*^*L*^*G*^(*T*^*G*^)^*T*^, *V*^*L*^ = *T*^*L*^*L*^*L*^(*T*^*L*^)^*T*^, ${\overset{*}{A}}^{GL}=T^{G}{\hat{A}}^{GL}{\left(T^{L}\right)}^{T}$, ${\overset{*}{A}}^{LG}=T^{L}{\hat{A}}^{LG}{\left(T^{G}\right)}^{T}$. Due to the specific diagonal structure of the matrices *D*^*LG*^, *D*^*GL*^ and Г, they remain unchanged after the transformation.

*Remark 1: Both matrices T*^*G*^ *and T*^*L*^ *can in turn be written as block-diagonal matrices with the number of blocks equal to the number of clusters in either layer. For our case*, *T*^*G*^ *is a matrix with l*_*G*_ *blocks and T*^*L*^ *is a matrix with l*_*L*_ *blocks such that T can be viewed as a block-diagonal matrix with a total of l blocks*.

If we define the vector **Ω**^*T*^ = [***ξ***^*T*^, ***κ***^*T*^], (12) and (13) can be recast as,

(14) $$\left[\begin{array}{ll} I_{n_{G}\times n_{G}} & 0_{n_{G}\times n_{L}}\\ 0_{n_{L}\times n_{G}} & 0_{n_{L}\times n_{L}} \end{array}\right]\ddot{\Omega }+\left[\begin{array}{cc} \Gamma & 0_{n_{G}\times n_{L}}\\ 0_{n_{L}\times n_{G}} & I_{n_{L}\times n_{L}} \end{array}\right]\dot{\Omega }\;-\left[\begin{array}{cc} V^{G}-D^{GL} & {\overset{*}{A}}^{GL}\\ {\overset{*}{A}}^{LG} & V^{L}-D^{LG} \end{array}\right]\Omega =\left[\begin{array}{c} {\text{r}}^{G}\\ {\text{r}}^{L} \end{array}\right],$$

which is in the familiar form,

(15) $$\tilde{M}\ddot{\Omega }+\tilde{C}\dot{\Omega }+\tilde{K}\Omega =\tilde{\text{f}}.$$

It is to be noted that the *n* × *n* matrix $\tilde{K}$, is block diagonal and is equal to the direct sum ${⊕}_{u=1}^{U}K_{u}$, where *K*_*u*_ is a (generally complex) *p*_*u*_ × *p*_*u*_ matrix with *p*_*u*_ the multiplicity of the *u*^*th*^ IRR in the permutation representation, *U* the number of IRRs present and *d*_*u*_ the dimension of the *u*^*th*^ IRR, so that ∑_*u*_ *d*_*u*_*p*_*u*_ = *n*. The matrix *T* contains information on which perturbations affecting different clusters get mapped to different IRRs [40]. Due to the block-diagonal structure of the matrix $\tilde{K}$, (14) can be decoupled into a number of lower-dimensional equations, where each equation corresponds to a block of the matrix $\tilde{K}$. There is one representation (labeled *u* = 1) which we call trivial and the associated block of the matrix $\tilde{K}$ corresponds to the dynamics of the quotient network.

*Definition 4 (Indicator Matrix): The n* × *l indicator matrix E has entries E*(*i*, *j*) = 1 *if node i belongs to the cluster j and 0 otherwise*,

*Definition 5 (Quotient Network): A Quotient Network is a reduced network where redundancies due to symmetries are removed. The dynamics of the quotient network is obtained by pre-multiplying* (10) *by* ((*E*^*T*^ *E*)^−1^*E*^*T*^), *yielding*,

(16) $$M_{q}\ddot{\hat{\text{X}}}+C_{q}\dot{\hat{\text{X}}}+K_{q}\hat{\text{X}}={\text{f}}_{\text{q}}$$

*where the n* × 1 *vectors* $\hat{\text{X}}(t)=\left({\left(E^{T}E\right)}^{-1}E^{T}\right)\text{X}(t)\;{\text{f}}_{\text{q}}=\left({\left(E^{T}E\right)}^{-1}E^{T}\right)\text{f}$, *and the matrices*

$$M_{q}=\left({\left(E^{T}E\right)}^{-1}E^{T}\right)ME,$$

$$C_{q}=\left({\left(E^{T}E\right)}^{-1}E^{T}\right)CE,$$

$$K_{q}=\left({\left(E^{T}E\right)}^{-1}E^{T}\right)KE.$$

Hence, the trivial representation is associated with all the clusters. However, it is possible that other IRR representations are only associated with some of the clusters (not all of them.) Each one of these other representations *u* > 1 describes the deviation/error dynamics of either an isolated cluster or a group of intertwined clusters [36]. A simple interpretation of isolated vs. intertwined clusters is the following. If a cluster is isolated, a perturbation affecting the power of any one of its nodes will not affect the deviation dynamics of other clusters. On the contrary, when a set of two or more clusters are intertwined, a perturbation affecting the power of any of the nodes in a cluster will affect the deviation dynamics of the remaining clusters in the set.

## III. DIAGONALIZATION

The quotient network provides a minimal representation of the full network. Our goal here is to describe the error dynamics between the full network dynamics and the quotient network dynamics.

(17) $$e(t)={\hat{X}}_{j}(t)-X_{i}(t)$$

where *X*_*i*_(t) is the dynamics of node *i* which belongs to cluster *j* of the entire network and ${\hat{X}}_{j}$ is the dynamics of node *j* of the quotient network, which corresponds to an average of the dynamics of all nodes in cluster *j*.

To quantify this error, we present an approach to diagonalize the blocks of the matrix $\tilde{K}$ with *u* > 1 and size *m* > 1. We consider an *m*-dimensional transverse block from (15), with dynamics,

(18) $$\hat{M}\ddot{\eta }+\hat{C}\dot{\eta }+\hat{K}\eta =\hat{\text{f}}$$

where $\hat{M}$ is an *m* × *m* diagonal block of the $\tilde{M}$ matrix, $\hat{C}$ is the corresponding *m* × *m* block of the $\tilde{C}$ matrix and $\hat{K}$ is the corresponding *m* × *m* block of the $\tilde{K}$ matrix. ***η*** and $\hat{\text{f}}$ are the corresponding *m* × 1 vectors from the **Ω** and $\tilde{\text{f}}$ vectors, respectively. In what follows, we will distinguish between three possible cases: 1) The block represents the error dynamics of intertwined clusters within the generator layer. This means that the $\hat{M}$ matrix will be a diagonal matrix with all non-zero elements in the main diagonal and the matrix $\hat{C}$ can either be *homogeneous*, i.e, it is a multiple of the identity matrix, or non-homogeneous. This is equivalent to a single layer network with either same or different damping coefficients which has already been studied in [5]. 2) The block represents the error dynamics of intertwined clusters within the load layer. This means that the $\hat{M}$ matrix is a zero matrix and the $\hat{C}$ matrix is the identity matrix. This is a simple case that allows a trivial decomposition. In this paper, we also consider a third, more complex case; 3) The block represents the error dynamics of intertwined clusters in different layers. This means that the $\hat{M}$ matrix is a diagonal matrix with both zero and non-zero elements along the main diagonal, i.e., it is a singular matrix different from the zero matrix. The matrix $\hat{C}$ can be non-homogeneous. To diagonalize such a system, we use the method described in [23]. The advantage of this method is that it is a direct generalization of the modal decoupling for the case that the $\hat{M}$ matrix is invertible, i.e, case 1. Should the system have an invertible mass matrix and be classically damped, i.e, satisfies the commutativity relationship, $\hat{C}{\hat{M}}^{-1}\hat{K}=\hat{K}{\hat{M}}^{-1}\hat{C}$ [8], [23], this method reduces to classical modal analysis. The method is summarized below. We consider the associated quadratic eigenvalue problem (QEP):

(19) $$\left(\hat{M}\lambda^{2}+\hat{C}\lambda +\hat{K}\right)\text{v}=0$$

The solution of this QEP yields a total of 2*m* eigenvalues, of which *σ* = *m* + *r* are finite and *ϵ* = *m* − *r* are *infinite*, where *r* is the rank of the matrix $\hat{M}$ and *m* is its size. We randomly assign 2*r* finite eigenvalues to conjugate pairs: *λ*_*i*_ and ${\hat{\lambda }}_{i}$ where *i* = 1, 2, 3, …, *r*, and we denote the remaining *ϵ* unpaired eigenvalues, called the “lone eigenvalues” by *μ* [23]. We now construct the matrices Λ and $\hat{\Lambda }$ which are diagonal matrices of dimension *r* × *r* and the matrix Ξ which is of dimension *ϵ* × *ϵ*. We also construct the matrices *V*, $\hat{V}$ and *W* whose columns are the eigenvectors of the matrices Λ, $\hat{\Lambda }$, and Ξ, respectively: **v**_*i*_, ${\hat{\text{v}}}_{i}$, *i* = 1, 2, 3, …, *r* and **w**_*i*_, *i* = 1, 2, 3, …, *ϵ*.

$$\Lambda =\overset{r}{\underset{i}{⊕}}\lambda_{i}\;\hat{\Lambda }=\overset{r}{\underset{i}{⊕}}\hat{\lambda_{i}}\;\Xi =\overset{\epsilon }{\underset{i}{⊕}}\mu_{i}$$

$$V=\left[{\text{v}}_{1}|\;\ldots \;|{\text{v}}_{r}\right]\;\hat{V}=\left[{\hat{\text{v}}}_{1}|\;\ldots \;|{\hat{\text{v}}}_{r}\right]\;W=\left[{\text{w}}_{1}|\;\ldots \;|{\text{w}}_{\epsilon }\right],$$

where here and in what follows, *C* = [*A*|*B*] symbolizes concatenation of two vectors/matrices with the same number of rows. We can now construct the diagonalized matrices:

$$\begin{array}{ccc} A_{2}=I_{r}⊕0_{\epsilon }, & A_{1}=(\Lambda +\hat{\Lambda })⊕-\Xi , & A_{0}=\Lambda \hat{\Lambda }⊕\Xi^{2}, \end{array}$$

In order to find consistent initial conditions, we also need to define,

$$\begin{array}{ccc} J_{xf}=J_{pf}=\Lambda ⊕\hat{\Lambda }⊕\Xi , & V_{xf}=[V|\hat{V}|W], & V_{pf}=\left[I_{r}|I_{r}\right]⊕I_{\epsilon }. \end{array}$$

Multiplying (19) by *β*^2^ = 1/*λ*^2^ yields:

(20) $$\left(\hat{M}+\hat{C}\beta +\hat{K}\beta^{2}\right)\text{v}=0.$$

The *ϵ* infinite eigenvalues from (19) correspond to *ϵ* zero eigenvalues in (20), *β* = 0. These *ϵ* zero eigenvalues and their corresponding eigenvectors must satisfy:

(21) $$\hat{M}V_{x,\infty }+\hat{C}V_{x,\infty }J_{x,\infty }+\hat{K}V_{x,\infty }J_{x,\infty }^{2}=0_{n\times \epsilon }$$

where *J*_*x*,∞_ is an order *ϵ* Jordan matrix of eigenvalues *β* = 0, and *V*_*x*,∞_ is is a *n* × *ϵ* matrix of the associated eigenvectors. Since (18) is nondefective, *J*_*x*,∞_ = 0_*ϵ*_, which implies $\hat{M}V_{x,\infty }=0_{n\times \epsilon }$. Therefore, *V*_*x*,∞_ is the matrix of eigenvectors in the null space of $\hat{M}$. Now, the transformation matrix *S* can be defined as,

(22) $$S=\left[\begin{array}{cc} V_{xf} & 0_{n\times \epsilon }\\ V_{xf}J_{xf} & V_{x,\infty } \end{array}\right]{\left[\begin{array}{cc} V_{pf} & 0_{n\times \epsilon }\\ V_{pf}J_{pf} & V_{p,\infty } \end{array}\right]}^{-1}$$

where *S* is a 2*m*-dimensional real orthogonal matrix, and *V*_*p*,∞_ is the matrix of eigenvectors in the null space of *A*_2_ (analogous to the case of $\hat{M}$ and *V*_*x*,∞_). If system (18) is written in the symmetric state space realization,

(23) $$\left[\begin{array}{cc} \hat{C} & \hat{M}\\ \hat{M} & 0 \end{array}\right]\left[\begin{array}{l} \dot{\eta }\\ \ddot{\eta } \end{array}\right]+\left[\begin{array}{cc} \hat{K} & \hat{0}\\ 0 & -\hat{M} \end{array}\right]\left[\begin{array}{l} \eta \\ \dot{\eta } \end{array}\right]=\left[\begin{array}{l} \hat{f}\\ 0 \end{array}\right],$$

the transformation *S* will produce the diagonal matrices *A*_0_, *A*_1_, and *A*_2_,

(24) $$S^{T}\left[\begin{array}{cc} \hat{C} & \hat{M}\\ \hat{M} & 0 \end{array}\right]S=\left[\begin{array}{cc} A_{1} & A_{2}\\ A_{2} & 0 \end{array}\right],\;S^{T}\left[\begin{array}{cc} \hat{K} & \hat{0}\\ 0 & -\hat{M} \end{array}\right]S=\left[\begin{array}{cc} A_{0} & 0\\ 0 & -A_{2} \end{array}\right].$$

We now derive the two transformation matrices:

(25) $$T_{1}=\left[(V\hat{\Lambda }-\hat{V}\Lambda ){\left(\hat{\Lambda }-\Lambda \right)}^{-1}|W\right]\;T_{2}=\left[(\hat{V}-V\Lambda ){\left(\hat{\Lambda }-\Lambda \right)}^{-1}|0_{n\times \epsilon }\right].$$

Using *T*_1_ and *T*_2_, we can calculate the transformed forcing function,

(26) $$\text{g}=T_{1}^{T}\hat{\text{f}}+T_{2}^{T}\dot{\hat{\text{f}}}.$$

The diagonalized equation in ***χ*** coordinates can be found using the *A*_0_, *A*_1_, *A*_2_ and **g** we have already calculated:

(27) $$A_{2}\ddot{\chi }+A_{1}\dot{\chi }+A_{0}\chi =\text{g}.$$

The initial conditions in the ***χ*** coordinates also need to be calculated from consistent initial conditions in the ***η*** coordinates,

(28) $$\left[\begin{array}{l} \chi (0)\\ \dot{\chi }(0) \end{array}\right]=S^{T}\left[\begin{array}{c} \eta (0)\\ \dot{\eta }(0)-V_{xf}Z_{xf}\text{f}(0) \end{array}\right]\;+\left[\begin{array}{c} 0\\ V_{pf}Z_{pf}\text{g}(0)+T_{2}^{T}\text{f}(0) \end{array}\right],$$

where the *σ* × *m* matrices *Z*_*xf*_, *Z*_*pf*_ are constructed as described in [23]. In what follows, without loss of generality we focus on the forced response and set the initial conditions in the *X*-coordinates to zero. Hence, we have zero initial conditions in the *η*-coordinates as well.

*Lemma 3: Consider zero initial conditions in X-coordinates. For unit step forcing* ${\hat{f}}_{i}$, *only the initial velocity for the second order modes are non-zero; all other initial conditions are zero*, *that is*, *if* **X**(0) = 0, $\dot{\text{X}}\left(0\right)=0$, *and* $\hat{\text{f}}(t)\in U$, *where* U *is the vector space for all unit step functions*, *then*,

$$\{\begin{array}{ll} \chi_{i}(0)=0 & \text{for }i=1,2,3\;\ldots \;n\\ {\dot{\chi }}_{i}(0)\neq 0 & \text{for }i=1,2,3,\;\ldots \;,n_{G}\\ {\dot{\chi }}_{i}(0)=0 & \text{for }i=n_{G}+1,n_{G}+2,\;\ldots \;,n. \end{array}$$

*Proof:* We know that if ${\hat{f}}_{i}\in U$, *f* (0) = 0 and ${\hat{f}}_{i}(0)\neq 0$. Also, we have 0 initial conditions in the *X* and *η* coordinates. So, we can calculate our modal initial conditions as:

(29) $$\left[\begin{array}{c} \chi (0)\\ \dot{\chi }(0) \end{array}\right]=\left[\begin{array}{c} 0\\ V_{pf}Z_{pf}\text{g}(0) \end{array}\right].$$

It is now obvious that *χ*_*i*_(0) = 0 ∀ *i*. Since the non zero term of (29)

$$\dot{\chi }(0)=V_{pf}Z_{pf}\text{g}(0),$$

it follows that $\text{g}=T_{2}^{T}\dot{\hat{\text{f}}}$ when $\hat{\text{f}}=0$ (from (26)). From (25), we can see that the last *ϵ* columns of *T*_2_ are zeros which means that the last *ϵ* rows of $T_{2}^{T}$ are zeros. Therefore,

$$T_{2}^{T}\dot{\hat{\text{f}}}(0)={\left[\chi_{1},\chi_{2},\;\ldots \;,\chi_{n_{G}},0,0,\;\ldots \;0\right]}^{T}$$

After pre-multiplying the expression by *V*_*pf*_ *Z*_*pf*_, we get the modal initial velocities of the system. This results in the last *ϵ* elements of $\dot{\chi }\left(0\right)$ to be zero which corresponds to the first order modes, thereby completing the proof. □

Note that we can use the transformation matrices to recover the displacements in *η* coordinates,

(30) $$\eta =T_{1}\chi +T_{2}\dot{\chi }-T_{3}\hat{\text{f}},\text{ where }T_{3}=T_{2}T_{2}^{T}.$$

We see that (27) can be broken into *m* independent equations, the solutions of which are called the ‘modes’ of the system.

(31) $${\left(A_{2}\right)}_{ii}{\ddot{\chi }}_{i}+{\left(A_{1}\right)}_{ii}{\dot{\chi }}_{i}+{\left(A_{0}\right)}_{ii}\chi_{i}=g_{i}.$$

*i* = 1, …, *m*. The linear combination of all of the modes, their derivatives, and the forcing function provides the solution for the error as shown in (30).

After diagonalization, the first *r* modes we obtain are second order modes, (*A*_2_)_*ii*_ ≠ 0, *i* = 1, …, *r*, and the remaining *m* − *r* modes are first order modes, (*A*_2_)_*ii*_ = 0, *i* = *r* + 1, …, *r* + *n*. In realistic systems with constant damping and inertia, it has been found that all modes are underdamped and propagate through the whole system with $\frac{D}{\lambda_{i}}<\gamma^{-1}$, ∀*i* > 1 [46], [47]. Reference [5] has shown that this assumption holds true even after the assumption of constant damping is removed. We can then characterize the modal responses in terms of well-known first order time responses and underdamped second order time responses to unit step forcing.

Each second order mode can be represented as,

(32) $${\ddot{\chi }}_{i}(t)+2\zeta_{i}\omega_{i}{\dot{\chi }}_{i}(t)+\omega_{i}^{2}\chi_{i}(t)=g_{i},\;i=1,2,\;\ldots \;,r,$$

where $\omega_{i}^{2}=A_{0ii}$ and *ζ*_*i*_ = (*A*_1_)_*ii*_/(2*ω*_*i*_). The solution to (32) can be written as,

(33) $$\chi_{i}(t)={\bar{\chi }}_{i}(t)+{\bar{\bar{\chi }}}_{i}(t),$$

where ${\bar{\chi }}_{i}(t)$ is the free evolution and ${\bar{\bar{\chi }}}_{i}(t)$ is the forced evolution. ${\bar{\chi }}_{i}(t)$ is given by:

(34) $${\bar{\chi }}_{i}(t)=e^{-\zeta_{i}\omega_{i}t}{\left(X_{m}\right)}_{i}\text{cos}\left(\omega_{i}\sqrt{1-\zeta_{i}^{2}}t-\psi_{i}\right)$$

where, ${\left(X_{m}\right)}_{i}=\sqrt{{\left(B_{1}\right)}_{i}^{2}+{\left(B_{2}\right)}_{i}^{2}}$ and $\psi_{i}={\text{tan}}^{-1}\left(\frac{{\left(B_{2}\right)}_{i}}{{\left(B_{1}\right)}_{i}}\right)$. (*B*_1_)_*i*_ and (*B*_2_)_*i*_ are constants that depend on the initial condition of the modes and can be calculated as follows:

$${\left(B_{1}\right)}_{i}=\chi_{i}(0)\;{\left(B_{2}\right)}_{i}=\frac{{\dot{\chi }}_{i}(0)+\zeta_{i}\omega_{i}\chi_{i}(0)}{\omega_{i}\sqrt{1-\zeta_{i}^{2}}}$$

We know from Lemma 3 that only ${\dot{\chi }}_{i}(0)$ are nonzero for second order modes. Therefore we have,

$${\left(B_{1}\right)}_{i}=0\;{\left(B_{2}\right)}_{i}=\frac{{\dot{\chi }}_{i}(0)}{\omega_{i}\sqrt{1-\zeta^{2}}}$$

We can use this into (34) and obtain,

(35) $${\bar{\chi }}_{i}(t)=\frac{{\dot{\chi }}_{i}(0)e^{-\zeta_{i}\omega_{i}t}}{\omega_{i}\sqrt{1-\zeta^{2}}}\text{sin}\left(\omega_{i}\sqrt{1-\zeta_{i}^{2}}t\right)$$

The underdamped forced solution is given by,

(36) $${\bar{\bar{\chi }}}_{i}(t)=\frac{g_{i}}{\omega_{i}^{2}}\left[1-\frac{e^{-\zeta_{i}\omega_{i}t}}{\sqrt{1-\zeta_{i}^{2}}}\text{sin}\left(\omega_{i}\sqrt{1-\zeta_{i}^{2}}t+{\text{cos}}^{-1\;}\zeta_{i}\right)\right],$$

*i* = 1, 2, …, *r*. The full response is the sum of (36) and (35).

On the other hand, each first order mode can be represented as,

(37) $${\dot{\chi }}_{i}(t)+\frac{\chi_{i}(t)}{\tau_{i}}={\hat{g}}_{i}(t)\;i=r+1,r+2,\;\ldots \;,m,$$

where, ${\hat{g}}_{i}(t)=\frac{g_{i}(t)}{{\left(A_{1}\right)}_{ii}}$ and $\tau_{i}=\frac{{\left(A_{1}\right)}_{ii}}{{\left(A_{0}\right)}_{ii}}$. The solution is equal to,

(38) $$\chi_{i}(t)=\tau_{i}{\hat{g}}_{i}(t)\left(1-e^{-t/\tau_{i}}\right)\;i=r+1,r+2,\;\ldots \;,m.$$

## IV. LINEAR COMBINATION OF MODES FOR MAXIMUM ERROR QUANTIFICATION

As stated in the previous section, for a unit step forcing, $\hat{\text{f}}$, the linear combination of the modes, their derivatives, and the forcing function can be used to calculate the deviations using (30), Here to ease this calculation, we introduce the concept of a ‘supermode.’

*Definition 6: A supermode is a linear combination of modal displacement and velocity. Supermodes can be of two types: First order supermode and second order supermode*.

Each supermode can be expressed by the equation,

(39) $${\hat{\chi }}_{ij}=\chi_{i}+\kappa_{ij}{\dot{\chi }}_{i},\;i,j=1,2,\;\ldots \;,m,$$

where *κ*_*ij*_ is a real constant. We note that ***η*** from (30) can be obtained as a linear combination of the ${\hat{\chi }}_{ij}$, *i*, *j* = 1, 2, …, *m*, from (39). Our definition of supermode in (39) is general, as *κ*_*ij*_ can be seen as a variable parameter that determines each supermode. In what follows we will see how first order supermodes and second order supermodes can be parameterized in a minimal number of parameters.

### A. FIRST ORDER SUPERMODES

First order supermodes, *i* = *r* + 1, *r* + 2, …, *m*, are obtained by plugging (38) in (39), yielding,

(40) $${\hat{\chi }}_{ij}=\tau_{i}{\hat{g}}_{i}+{\hat{g}}_{i}e^{-\frac{t}{\tau_{i}}}\left(\kappa_{ij}-\tau_{i}\right),$$

which converges to the steady state,

(41) $${\hat{\chi }}_{ij}^{SS}=\tau_{i}{\hat{g}}_{i}.$$

It can be seen from (40) that a first order supermode is completely parameterized by the constant (*κ*_*ij*_), modal forcing (${\hat{g}}_{i}$) and time constant (*τ*_*i*_).

### B. SECOND-ORDER SUPERMODES

Second order supermodes, *i* = 1, 2, 3 …, *r*, are obtained by plugging (36) in (39). We take the first derivative of the solution to get,

$${\dot{\chi }}_{i}=\frac{e^{-\zeta_{i}\omega_{i}t}}{\varrho_{i}}\left[g_{i}\text{ sin }\omega_{i}\varrho_{i}t+{\dot{\chi }}_{i}(0)\left(\varrho_{i}\text{ cos }\varrho_{i}t-\zeta_{i}\omega_{i}\text{ sin }\varrho_{i}t\right)\right]$$

where $\varrho_{i}=\omega_{i}\sqrt{1-\zeta_{i}^{2}}$. So, we can simplify (39) as follows,

(42) $${\hat{\chi }}_{ij}=\frac{e^{-\zeta_{i}\omega_{i}t}}{\varrho_{i}}\left[Q_{ij}^{\left[1\right]}\text{ sin }\varrho_{i}t+Q_{ij}^{\left[2\right]}\text{ cos }\varrho_{i}t\right]+\frac{g_{i}}{\omega_{i}^{2}},$$

where,

$$Q_{ij}^{\left[1\right]}=\left(\kappa_{ij}-\frac{\zeta_{i}}{\omega_{i}}\right)g_{i}+\left(1-\zeta_{i}\omega_{i}\kappa_{ij}\right){\dot{\chi }}_{i}(0),$$

$$Q_{ij}^{\left[2\right]}=\left(\kappa_{ij}{\dot{\chi }}_{i}(0)-\frac{g_{i}}{\omega_{i}^{2}}\right)\varrho_{i}$$

This converges to a steady state

(43) $${\hat{\chi }}_{ij}^{SS}=\frac{g_{i}}{\omega_{i}^{2}}.$$

The peak time can be calculated by setting ${\dot{\hat{\chi }}}_{i}(t)=0$,

(44) $${\dot{\hat{\chi }}}_{ij}(t)=\frac{-\zeta_{i}\omega_{i}e^{-\zeta_{i}\omega_{i}t}}{\rho_{i}}\left[Q_{ij}^{\left[1\right]}\text{ sin }\rho_{i}t+Q_{ij}^{\left[2\right]}\text{ cos }\rho_{i}t\right]\;+\frac{e^{-\zeta_{i}\omega_{i}t}}{\rho_{i}}\left[\rho_{i}Q_{ij}^{\left[1\right]}\text{ cos }\rho_{i}t-\rho_{i}Q_{ij}^{\left[2\right]}\text{ sin }\rho_{i}t\right],$$

therefore, all the local extrema of the supermode are at,

(45) $$t_{ij}^{\text{p}}=\left({\text{tan}}^{-1}\left[\frac{Q_{ij}^{\left[1\right]}\varrho_{i}-\zeta_{i}\omega_{i}Q_{ij}^{\left[2\right]}}{Q_{ij}^{\left[2\right]}\varrho_{i}+\zeta_{i}\omega_{i}Q_{ij}^{\left[1\right]}}\right]+k\pi \right)\frac{1}{\varrho_{i}},\;k=0,1,\;\ldots \;$$

which can be plugged into (42) to find the associated supermode peak value, ${\hat{\chi }}_{ij}^{\text{p}}$. Since there is damping in the system, one may expect the first peak to always be the global maximum. However, it is also possible that this local maximum point is preceded by a local minimum point. So it becomes necessary to check (45) for *k* = 0, 1, to determine the supermodal peak.

By calculating ${\dot{\hat{\chi }}}_{ij}(0)$,

(46) $${\dot{\hat{\chi }}}_{ij}(0)=\rho_{i}Q_{ij}^{\left[1\right]}-\zeta_{i}\omega_{i}Q_{ij}^{\left[2\right]},$$

an analytical condition can be found to determine the supermodal peak. If ${\dot{\hat{\chi }}}_{ij}(0)$ is positive (negative), then the first (second) peak is the supermodal peak. If instead ${\dot{\hat{\chi }}}_{ij}(0)=0$, the curvature of the supermode at *t* = 0 should be assessed,

(47) $${\ddot{\hat{\chi }}}_{ij}(t)=\frac{\zeta_{i}^{2}\omega_{i}^{2}e^{-\zeta_{i}\omega_{i}t}}{\rho_{i}}\left[Q_{ij}^{\left[1\right]}\text{ sin }\rho_{i}t+Q_{ij}^{\left[2\right]}\text{ cos }\rho_{i}t\right]\;+\frac{-2\zeta_{i}\omega_{i}e^{-\zeta_{i}\omega_{i}t}}{\rho_{i}}\left[\rho_{i}Q_{ij}^{\left[1\right]}\text{ cos }\rho_{i}t-\rho_{i}Q_{ij}^{\left[2\right]}\text{ sin }\rho_{i}t\right]\;+\frac{e^{-\zeta i\omega_{i}t}}{\rho_{i}}\left[-\rho_{i}^{2}Q_{ij}^{\left[1\right]}\text{ sin }\rho_{i}t-\rho_{i}^{2}Q_{ij}^{\left[2\right]}\text{ cos }\rho_{i}t\right].$$

Since ${\dot{\hat{\chi }}}_{ij}(0)=Q_{ij}^{\left[1\right]}\rho_{i}-\zeta_{i}\omega_{i}Q_{ij}^{\left[2\right]}=0$,

(48) $${\ddot{\hat{\chi }}}_{ij}(0)=-\left(\zeta_{i}\omega_{i}Q_{ij}^{\left[1\right]}+\rho_{i}Q_{ij}^{\left[2\right]}\right).$$

Therefore, if the supermode is convex at $t=0\left({\ddot{\hat{\chi }}}_{ij}(0)>0\right)$, the next peak corresponding to *k* = 1 is the global peak. If the supermode is concave at $t=0\left({\ddot{\hat{\chi }}}_{ij}(0)<0\right)$, the peak corresponding to *k* = 2 is the global peak. In summary, the global peak is found at,

(49) $$k=\{\begin{array}{ll} 0, & \text{if }{\dot{\hat{\chi }}}_{ij}(0)>0,\\ 1, & \text{if}\{\begin{array}{ll} {\dot{\hat{\chi }}}_{ij}(0)<0, & \text{OR}\\ {\dot{\hat{\chi }}}_{ij}(0)=0, & {\ddot{\hat{\chi }}}_{ij}(0)>0, \end{array}\\ 2, & \text{if }{\dot{\hat{\chi }}}_{ij}(0)=0,\;{\ddot{\hat{\chi }}}_{ij}(0)<0, \end{array}$$

where ${\dot{\hat{\chi }}}_{ij}(0)$ and ${\ddot{\hat{\chi }}}_{ij}(0)$ are defined in (46) and (48), respectively.

Equation (42) shows that a second order supermode can be completely parameterized by its constant (*κ*_*ij*_), natural frequency (*ω*_*i*_), damped frequency (*ζ*), modal forcing (*g*_*i*_), and initial condition (*χ*_*i*_(0)).

### C. LINEAR COMBINATION OF SUPERMODES

Each *η*_*i*_ can be written as a linear combination of supermodes and of the forcing function, ${\hat{f}}_{i}$,

(50) $$\eta_{i}=\sum_{j=1}^{m}\left(C_{ij}^{\left[1\right]}{\hat{\chi }}_{ij}-C_{ij}^{\left[2\right]}{\hat{f}}_{i}\right)$$

in the coefficients $C_{ij}^{\left[1\right]}$ and $C_{ij}^{\left[2\right]}$ which denote how much a certain supermode affects the error dynamics. We note that *κ*_*ij*_ from (39) and $C_{ij}^{\left[1\right]}$, and $C_{ij}^{\left[2\right]}$ from (50) are variable constants that can be arbitrarily assigned. These constants can be chosen based on the specific purpose of the analysis. For example, one can pick *κ*_*ij*_ = *T*_2_(*i*, *j*)/*T*_1_(*i*, *j*), $C_{ij}^{\left[1\right]}=T_{1}(i,j)$, and $C_{ij}^{\left[2\right]}=T_{3}(i,j)$, where *i*, *j* = 1, …, *m*, to obtain ***η*** from (30).

Since the expressions for steady state values of the supermodes have been derived, we can calculate the steady state error as,

(51) $$\eta_{i}^{SS}=\sum_{j=1}^{m}\left(C_{ij}^{\left[1\right]}{\hat{\chi }}_{ij}^{SS}-C_{ij}^{\left[2\right]}{\hat{f}}_{i}\right)$$

For peak times, we use a slightly modified version of the technique described in [5]. First we take the sum of the supermodal peak values and non-transformed forcing functions for all supermodes. We call this *η*^L^,

(52) $$\eta^{\text{L}}={\hat{\chi }}_{ij}^{\text{p}}-C_{ij}^{\left[2\right]}{\hat{f}}_{i},$$

For first order supermodes, the peak is given by ${\hat{\chi }}_{ij}^{\text{p}}={\hat{\chi }}_{ij}^{SS}$, *i* = *r* + 1, …, *m*, and the peak time can be approximated with the settling time equal to $t_{ij}^{\text{p}}=4\tau_{i}$. The *η*^L^s calculated from (52) are then cumulatively added in ascending order of peak time and the peak time corresponding to the largest of these cumulatively added values is then used as the initial guess in the equation:

$$\sum_{j=1}^{m}{\dot{\hat{\chi }}}_{ij}=0$$

which can be expanded into:

(53) $$\sum_{i=1}^{r}\frac{e^{-\zeta_{i}\omega_{i}t}}{\varrho_{i}}\left[{\hat{Q}}_{ij}^{\left[2\right]}\text{ cos }\varrho_{i}t-{\hat{Q}}_{ij}^{\left[1\right]}\text{ sin }\varrho_{i}t\right]\;+\sum_{i=r+1}^{m}\frac{\tau_{i}-\kappa_{ij}}{\tau_{i}}{\hat{g}}_{i}e^{-\frac{t}{\tau_{i}}}=0$$

where,

$${\hat{Q}}_{ij}^{\left[1\right]}=\zeta_{i}\omega_{i}Q_{ij}^{\left[1\right]}+\varrho_{i}Q_{ij}^{\left[2\right]}\text{ and }{\hat{Q}}_{ij}^{\left[2\right]}=\varrho_{i}Q_{ij}^{\left[1\right]}-\zeta_{i}\omega_{i}Q_{ij}^{\left[2\right]}.$$

Solving (53) numerically using our calculated initial time provides the time for the peak error $t_{i}^{\text{peak}}$. It is also important to note that the closer the peak times are, the more accurate this initial guess is and the farther apart they are, the more iterations will be required to converge to the actual solution. Once the peak time is known, it can be plug back into (50) to find the peak error.

(54) $$\eta_{i}^{\text{peak}}=\sum_{j=1}^{m}\left({\hat{\chi }}_{ij}\left(t_{i}^{\text{peak}}\right)-C_{ij}^{\left[2\right]}{\hat{f}}_{i}\right).$$

Finally, the maximum error could either be the peak error (if the second order supermode dominates) or the steady state error (if the first order supermode dominates). Thus, we take the largest value between $\eta_{i}^{SS}$ and $\eta_{i}^{\text{peak}}$ as the maximum error $\eta_{i}^{\text{max}}$,

(55) $$\eta_{i}^{\text{max}}=\text{max}\left(\eta_{i}^{SS},\eta_{i}^{\text{peak}}\right)$$

## V. CASE STUDIES

In this section we provide 2 examples to demonstrate our method: Example 1 shows how our method can be utilized to find the maximum deviation error for a small network and example 2 demonstrates application of the method to the case of the IEEE 145-bus test grid.

### A. EXAMPLE 1

**FIGURE 1.**
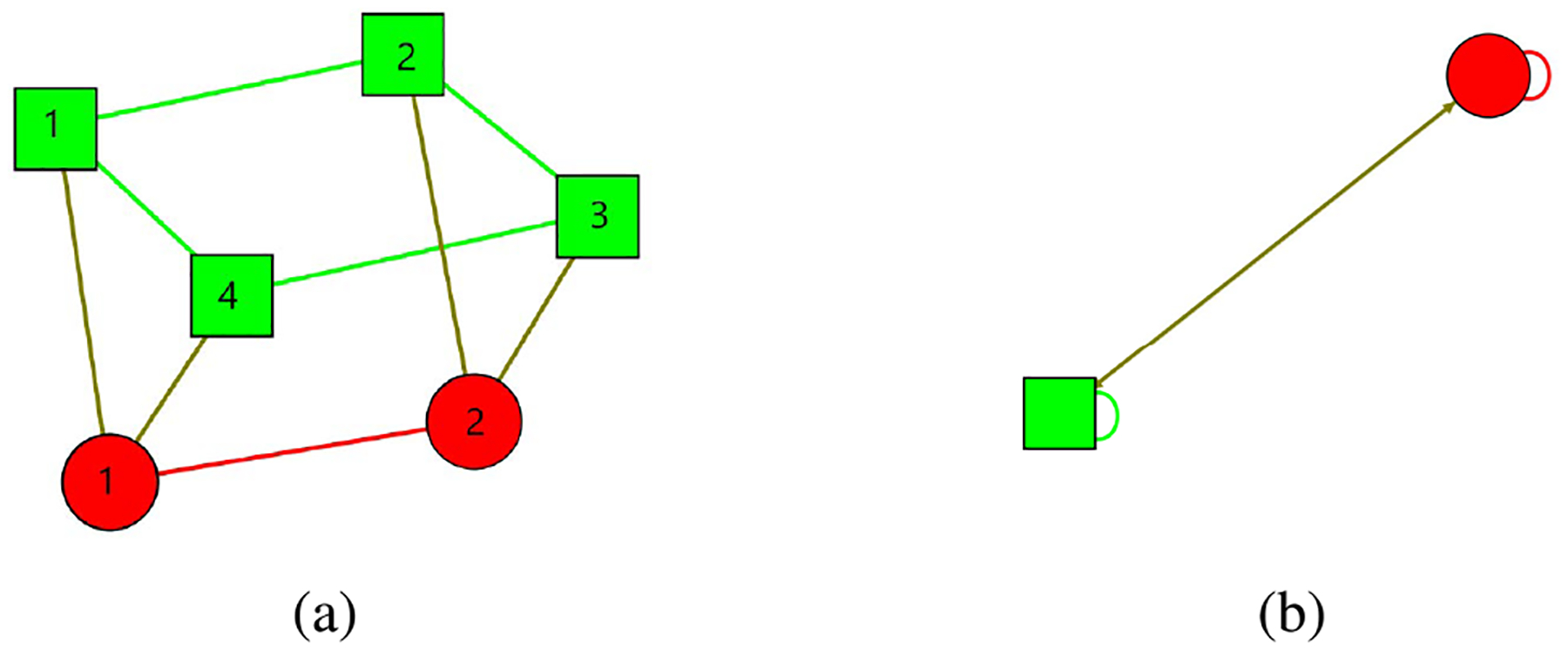
*(a)*: Full network, *(b)*: Quotient network. Generators are represented by green squares and loads are represented as red circles.

Our first example is the network in Figure 1 with *n*_*G*_ = 4 nodes in the generator layer and *n*_*L*_ = 2 nodes in the load layer, with **p**^*G*^ = [0.1 0.1 0.1 0.2]^*T*^ and **p**^*L*^ = [−0.2 −0.3]^*T*^.

The network dynamics is given by the following equation:

(56) $$\left[\begin{array}{llllll} 1 & 0 & 0 & 0 & 0 & 0\\ 0 & 1 & 0 & 0 & 0 & 0\\ 0 & 0 & 1 & 0 & 0 & 0\\ 0 & 0 & 0 & 1 & 0 & 0\\ 0 & 0 & 0 & 0 & 0 & 0\\ 0 & 0 & 0 & 0 & 0 & 0 \end{array}\right]\ddot{\text{X}}+\left[\begin{array}{cccccc} 0.9 & 0 & 0 & 0 & 0 & 0\\ 0 & 0.9 & 0 & 0 & 0 & 0\\ 0 & 0 & 0.9 & 0 & 0 & 0\\ 0 & 0 & 0 & 0.9 & 0 & 0\\ 0 & 0 & 0 & 0 & 1 & 0\\ 0 & 0 & 0 & 0 & 0 & 1 \end{array}\right]\dot{\text{X}}\;-\left[\begin{array}{cccccc} -3 & 1 & 0 & 1 & 1 & 0\\ 1 & -3 & 1 & 0 & 0 & 1\\ 0 & 1 & -3 & 1 & 0 & 1\\ 1 & 0 & 1 & -3 & 1 & 0\\ 1 & 0 & 0 & 1 & -3 & 1\\ 0 & 1 & 1 & 0 & 1 & -3 \end{array}\right]\text{X}=\left[\begin{array}{c} 0.1\\ 0.1\\ 0.1\\ 0.2\\ -0.2\\ -0.3 \end{array}\right].$$

Using the IRR transformation matrix *T*,

$$T=\left[\begin{array}{cccccc} 0.5 & 0.5 & 0.5 & 0.5 & 0 & 0\\ 0 & 0 & 0 & 0 & \frac{1}{\sqrt{2}} & \frac{1}{\sqrt{2}}\\ 0 & 0 & 0 & 0 & \frac{1}{\sqrt{2}} & -\frac{1}{\sqrt{2}}\\ 0.5 & -0.5 & -0.5 & 0.5 & 0 & 0\\ 0.5 & 0.5 & -0.5 & -0.5 & 0 & 0\\ 0.5 & -0.5 & 0.5 & -0.5 & 0 & 0 \end{array}\right],$$

(56) is transformed into,

(57) $$\left[\begin{array}{llllll} 1 & 0 & 0 & 0 & 0 & 0\\ 0 & 0 & 0 & 0 & 0 & 0\\ 0 & 0 & 0 & 0 & 0 & 0\\ 0 & 0 & 0 & 1 & 0 & 0\\ 0 & 0 & 0 & 0 & 1 & 0\\ 0 & 0 & 0 & 0 & 0 & 1 \end{array}\right]\ddot{\eta }+\left[\begin{array}{cccccc} 0.9 & 0 & 0 & 0 & 0 & 0\\ 0 & 1.0 & 0 & 0 & 0 & 0\\ 0 & 0 & 1.0 & 0 & 0 & 0\\ 0 & 0 & 0 & 0.9 & 0 & 0\\ 0 & 0 & 0 & 0 & 0.9 & 0\\ 0 & 0 & 0 & 0 & 0 & 0.9 \end{array}\right]\dot{\eta }\;-\left[\begin{array}{cccccc} -1 & \sqrt{2} & 0 & 0 & 0 & 0\\ \sqrt{2} & -2 & 0 & 0 & 0 & 0\\ 0 & 0 & -4 & \sqrt{2} & 0 & 0\\ 0 & 0 & \sqrt{2} & -3 & 0 & 0\\ 0 & 0 & 0 & 0 & -3 & 0\\ 0 & 0 & 0 & 0 & 0 & -5 \end{array}\right]\eta =\left[\begin{array}{c} 0.2500\\ -0.3536\\ 0.0707\\ 0.0500\\ -0.0500\\ -0.0500 \end{array}\right]$$

The 2 × 2 diagonal block on the top left of the $\hat{K}$ matrix of (57) corresponds to the quotient network dynamics. The quotient network is depicted in Figure 1(b). The other diagonal blocks represent the error dynamics between the full network and the quotient network, which is what we are interested in. The rightmost 2 blocks are simple so we diagonalize the second 2 × 2 diagonal block from the left to demonstrate our method. The dynamics of this one block is,

(58) $$\left[\begin{array}{ll} 0 & 0\\ 0 & 1 \end{array}\right]\ddot{\eta }+\left[\begin{array}{cc} 1 & 0\\ 0 & 0.9 \end{array}\right]\dot{\eta }+\left[\begin{array}{cc} 4 & -\sqrt{2}\\ -\sqrt{2} & 3 \end{array}\right]\eta =\left[\begin{array}{l} 0.0707\\ 0.0500 \end{array}\right]$$

The 3 finite eigenvalues for this 2 × 2 system are, *λ*_1_ = −0.5193 + 1.5232*i*, *λ*_2_ = −0.5193 − 1.5232*i* and *λ*_3_ = −3.8615. Pairing *λ*_1_ and *λ*_2_ as conjugates and *λ*_3_ as the lone eigenvalue, we can find the normalized eigenvectors,

$${\text{v}}_{\infty }=\left[\begin{array}{l} 1\\ 0 \end{array}\right]\;{\text{v}}_{1}=\left[\begin{array}{l} 0.3457-0.1544i\\ 1.0170-0.0077i \end{array}\right]\;{\text{v}}_{2}=\left[\begin{array}{l} 0.3457+0.1544i\\ 1.0170+0.0077i \end{array}\right]\;\text{w}=\left[\begin{array}{l} 2.0327\\ 0.1991 \end{array}\right].$$

We can now compute the matrices Λ, $\hat{\Lambda }$, *V*, $\hat{V}$, Ξ and *W*,

$$\Lambda =[-0.5193+1.5232i]\;V=\left[\begin{array}{l} 0.3457-0.1544i\\ 1.0170-0.0077i \end{array}\right],$$

$$\hat{\Lambda }=[-0.5193-1.5232i]\;\hat{V}=\left[\begin{array}{l} 0.3457+0.1544i\\ 1.0170+0.0077i \end{array}\right],$$

$$\Xi =[-3.8615],\;W=\left[\begin{array}{l} 2.0327\\ 0.1991 \end{array}\right].$$

We then solve for *T*_1_, *T*_2_, and *T*_3_,

$$T_{1}=\left[\begin{array}{ll} 0.2930 & 2.0327\\ 1.0144 & 0.1991 \end{array}\right]\;T_{2}=\left[\begin{array}{ll} -0.1014 & 0.0000\\ -0.0050 & 0.0000 \end{array}\right]\;T_{3}=\left[\begin{array}{ll} 0.0103 & 0.0005\\ 0.0005 & 0.0000 \end{array}\right].$$

We then calculate the decoupled coefficient matrices *A*_2_, *A*_1_, *A*_0_, transformed power **g**, and initial condition ***χ***(0),

$$A_{2}=\left[\begin{array}{ll} 1 & 0\\ 0 & 0 \end{array}\right],\;A_{1}=\left[\begin{array}{ll} 1.0385 & 0.0000\\ 0.0000 & 3.8615 \end{array}\right],\;\text{g}=\left[\begin{array}{l} 0.0714\\ 0.1537 \end{array}\right],\;A_{0}=\left[\begin{array}{cc} 2.5897 & 0.0000\\ 0.0000 & 14.9108 \end{array}\right],\;\chi (0)=\left[\begin{array}{c} 0\\ 0\\ -0.0074\\ 0 \end{array}\right],$$

resulting in the following decoupled equation,

(59) $$\left[\begin{array}{ll} 1 & 0\\ 0 & 0 \end{array}\right]\ddot{\chi }+\left[\begin{array}{ll} 1.0385 & 0.0000\\ 0.0000 & 3.8615 \end{array}\right]\dot{\chi }\;+\left[\begin{array}{cc} 2.5897 & 0.0000\\ 0.0000 & 14.9108 \end{array}\right]\chi =\left[\begin{array}{l} 0.0714\\ 0.1537 \end{array}\right]$$

Equation (59) describes the dynamics of two supermodes, a second order supermode (*i* = 1) and a first order supermode (*i* = 2). To calcuate the supermodal peak for each, we extract some parameters from the diagonalized system: *ζ*_1_ = 0.3227, *ω*_1_ = 1.6093, $\tau_{2}=\frac{1}{3.8615}$, ${\hat{g}}_{2}=0.0398$.

**FIGURE 2.**
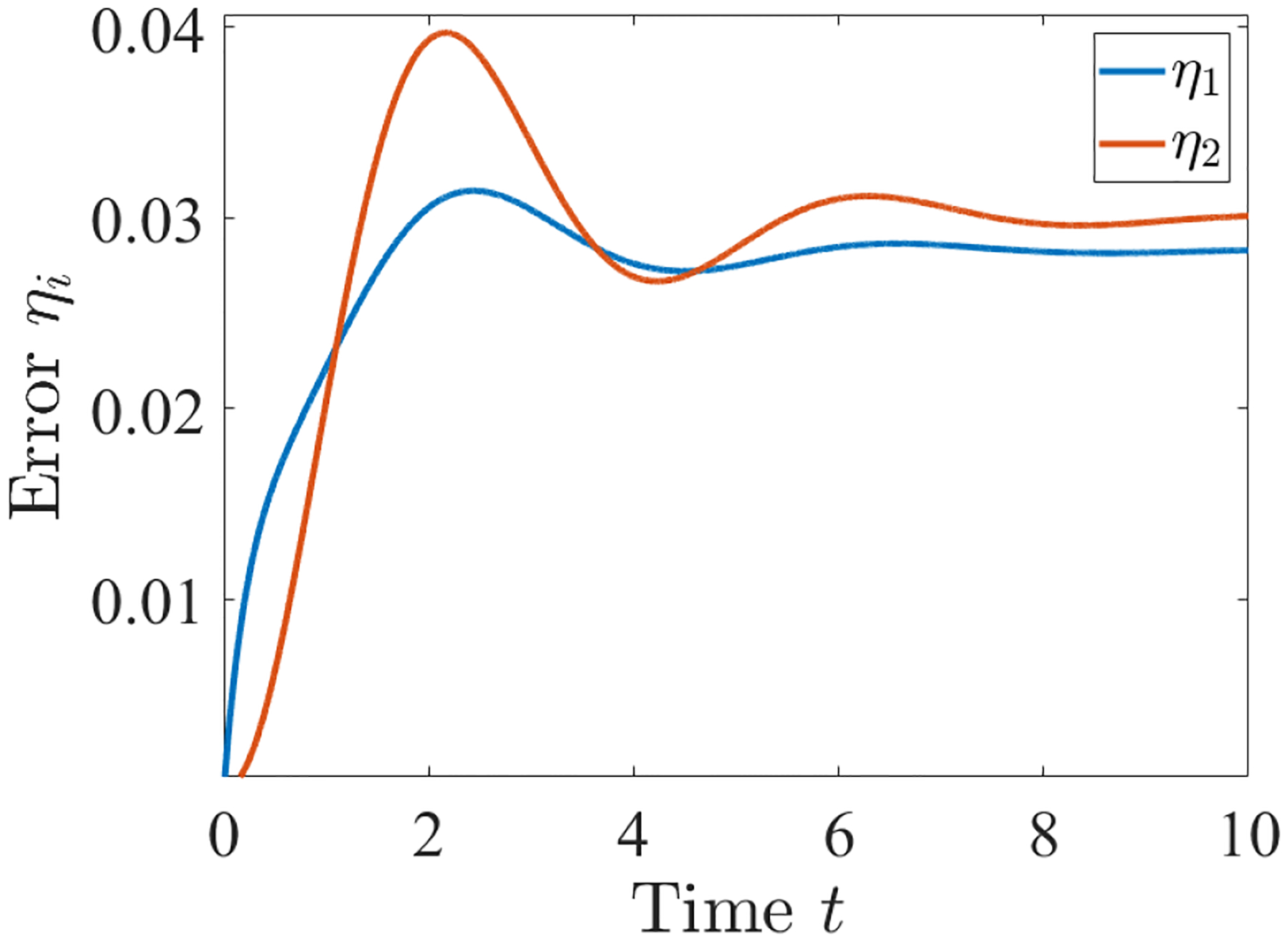
Error vs. time for the network in figure 1. Each curve represents the error dynamics due to the power not respecting the symmetries in the two layers of the network.

Table 1 provides information on the supermodal peak time and associated peak calculated via our method and compares it to the actual peak time and peak obtained by solving the linear ODE. We also cumulatively add the peaks to calculate the initial guess. Table 2 provides information on the maximum error values for our system calculated using our approach and compares it to the error values obtained by directly solving the linear ODE (rightmost column of the Table.) This is also consistent with the plot of the error dynamics shown in Fig. 2.

Using the IRR transformation matrix we find how this error corresponds to the dynamics of the full network and the quotient network: $\eta_{1}=\sqrt{2}\left({\hat{X}}_{2}-X_{6}\right)$ and $\eta_{2}=2{\hat{X}}_{1}-\left(X_{2}+X_{3}\right)$ where ${\hat{X}}_{1}=\frac{X_{1}+X_{2}+X_{3}+X_{4}}{4}$ and ${\hat{X}}_{2}=\frac{X_{5}+X_{6}}{2}$ are the dynamics of the two nodes of the associated quotient network.

### B. EXAMPLE 2

**FIGURE 3.**
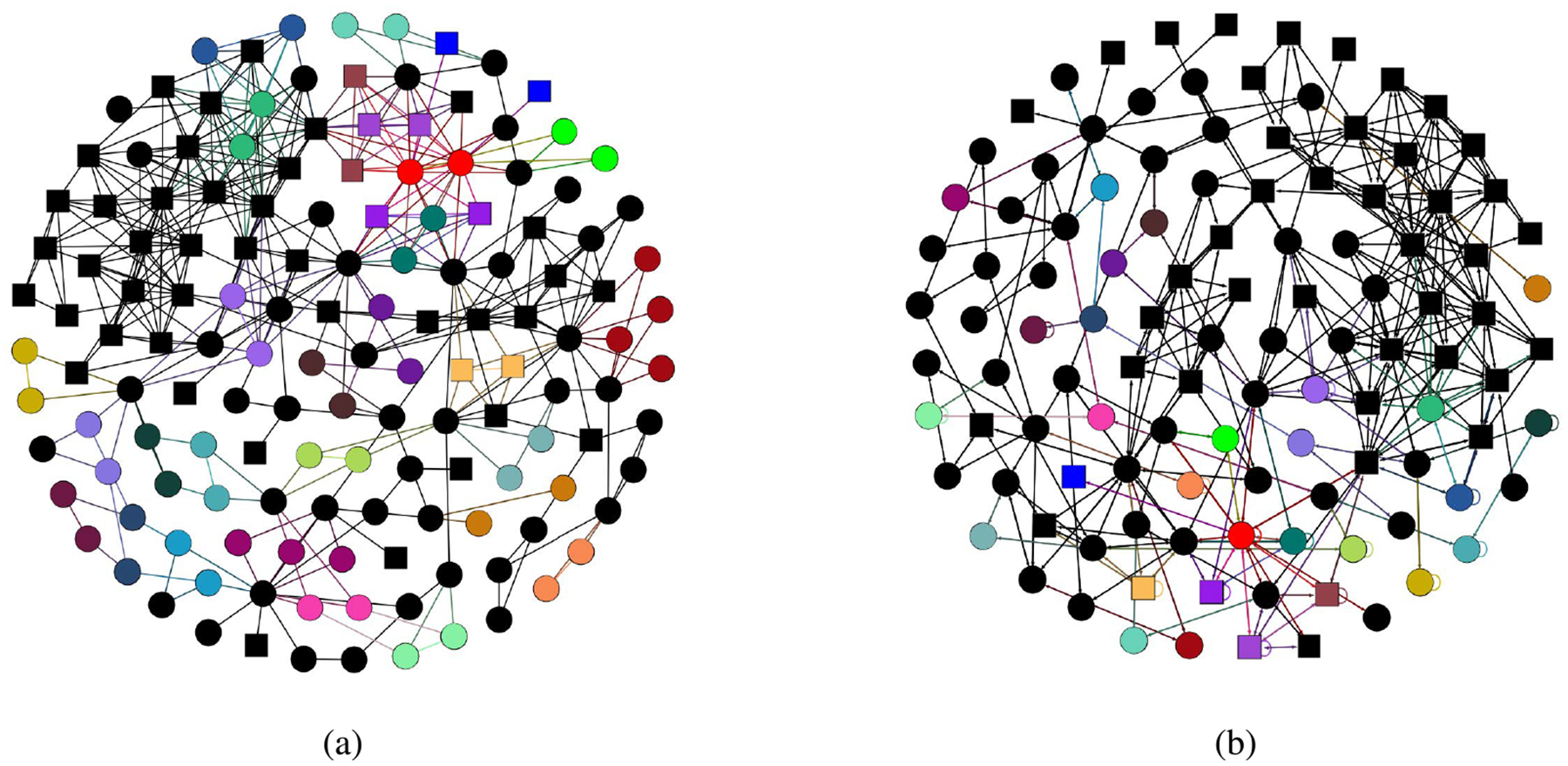
*(a)* Full network representation of IEEE145 test grid. *(b)* Quotient network representation of IEEE145 test grid. Circles indicate load nodes/clusters and squares indicate generator nodes/clusters. Nodes belonging to the same clusters are colored the same in (a) and the same colors are used for the clusters shown in (b). Nodes colored black belong to a trivial cluster.

Our second example is an application to the IEEE145 bus network with *n*_*G*_ = 50 generator nodes, *n*_*L*_ = 95 load nodes, and 422 transmission lines [50]. The network is shown in Fig. 3a and the corresponding quotient network in Fig. 3b. We assume that the power produced by the generator nodes is equal to $q_{i}^{G}=0.2$ and the power absorbed by the load nodes is equal $q_{i}^{L}=-0.1053$.

Using the IRR transformation, we obtain twentyfour transverse blocks, of which nineteen are scalar, three are 2 × 2, one is 3 × 3 and one is 4 × 4. These blocks represent the deviation dynamics of the individual nodes compared to their clusters. To demonstrate our method, we choose the 3 × 3 blocks, which corresponds to the error dynamics of three intertwined clusters, with two nodes each. The first two clusters are formed of load nodes {121, 122} and {76, 81}, while the third cluster is formed of generator nodes {21, 22}. These three clusters can be seen in Figure 3, where their nodes are represented as red circles, green circles and blue squares on the top right of Fig. 3a, respectively. They map to similarly colored and shaped nodes of the quotient network, all shown in the bottom left of Fig. 3b.

In order to characterize the error dynamics, we increase the power of node 76 to −0.2 to induce an asymmetry in power within that cluster. We also increase the power of node 22 to 0.5053 in order to maintain the network balance. Since nodes in the same clusters do not have the same power, it is expected that the quotient network dynamics will not represent the full network dynamics, which results in the aforementioned error dynamics. As in the previous examples, we are interested in characterizing the maximum overshoot of the error dynamics. Our chosen 3 × 3 block is associated with the following system of equations:

(60) $$\left[\begin{array}{lll} 0 & 0 & 0\\ 0 & 0 & 0\\ 0 & 0 & 1 \end{array}\right]\ddot{\eta }+\left[\begin{array}{ccc} 1 & 0 & 0\\ 0 & 1 & 0\\ 0 & 0 & 0.9 \end{array}\right]\dot{\eta }+\left[\begin{array}{ccc} 19 & -1 & -1\\ -1 & 2 & 0\\ -1 & 0 & 1 \end{array}\right]\eta \;=\left[\begin{array}{c} 0\\ 0.0670\\ 0.2159 \end{array}\right]$$

Using our method, we diagonalize this system,

(61) $$\left[\begin{array}{lll} 1 & 0 & 0\\ 0 & 0 & 0\\ 0 & 0 & 0 \end{array}\right]\ddot{\eta }+\left[\begin{array}{ccc} 0.9040 & 0 & 0\\ 0 & 1.9402 & 0\\ 0 & 0 & 19.0557 \end{array}\right]\dot{\eta }\;+\left[\begin{array}{ccc} 19 & -1 & -1\\ -1 & 2 & 0\\ -1 & 0 & 1 \end{array}\right]\eta =\left[\begin{array}{c} 0.2171\\ 0.0991\\ -0.0144 \end{array}\right]$$

We also obtain the transformation matrices and initial condition:

$$T_{1}=\left[\begin{array}{ccc} 0.0534 & 0.0832 & 4.3585\\ 0.0174 & 1.3912 & -0.2555\\ 1.0003 & 0.0276 & 0.0126 \end{array}\right],\;T_{2}=\left[\begin{array}{lll} -0.0040 & 0 & 0\\ -0.0196 & 0 & 0\\ -0.0002 & 0 & 0 \end{array}\right],\;T_{3}=10^{-03}\times \left[\begin{array}{lll} 0.0163 & 0.0791 & 0.0008\\ 0.0791 & 0.3833 & 0.0039\\ 0.0008 & 0.0039 & 0.0000 \end{array}\right],\;\chi (0)=\left[\begin{array}{c} 0\\ 0\\ 0\\ -0.0014\\ 0\\ 0 \end{array}\right].$$

**FIGURE 4.**
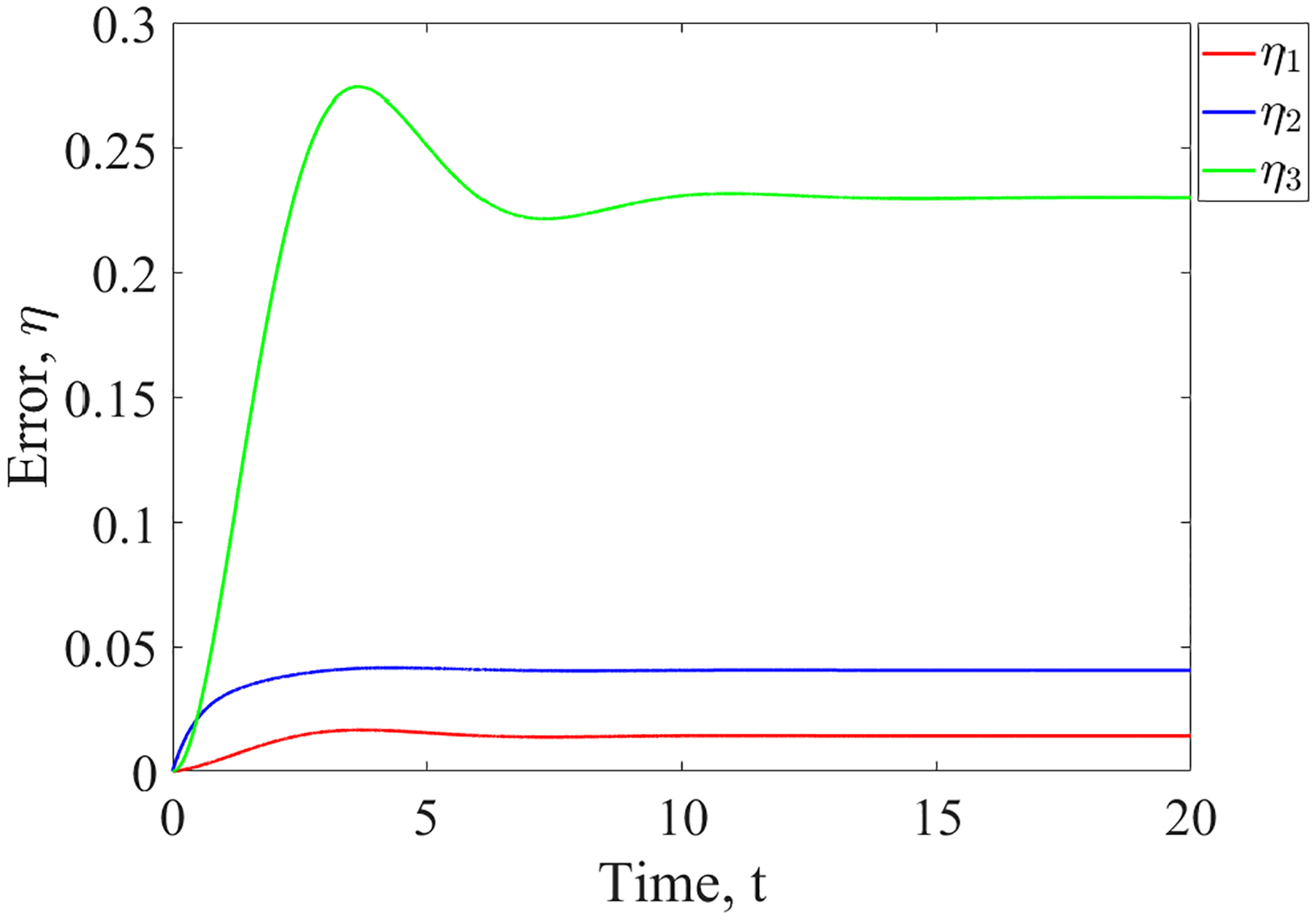
Error vs. time for the network in figure 3. Each curve represents the error dynamics in the 3 intertwined clusters presented in example 3.

Using parameters taken from the diagonalized system and applying the same method as in the previous example, we can calculate the initial guess for each *η*, which is shown for *η*_1_ in Table 3. This initial guess is then used to compute the maximum errors shown in Table 4. The values in the tables are consistent with the error dynamics shown in Fig. 4.

Like in the previous examples, the *η*s can be expressed in terms of displacements of nodes of the full network and the quotient network. $\eta_{1}=\sqrt{2}\left({\hat{X}}_{95}-X_{211}\right)$, $\eta_{2}=\sqrt{2}\left({\hat{X}}_{20}-X_{76}\right)$, $\eta_{3}=\sqrt{2}\left({\hat{X}}_{20}-X_{21}\right)$, where ${\hat{X}}_{i}$ represents the displacement of the *i*^*th*^ quotient node (the average of the displacements of the individual nodes in that cluster), and *X*_*i*_ is the displacement of node *i* of the full network.

## VI. CONCLUSION

In this paper, we have specialized the structure-preserving model of the swing equation to a multi-layer network with two layers, one formed of generator nodes and one formed of motor nodes. In the presence of symmetries, a lower dimensional model for the dynamics is provided by the so-called quotient network. However, such representation is often inexact when nodes in the same cluster generate or consume different amounts of power, which makes it important to characterize how much the actual network dynamics deviates from that of the quotient network.

A main difference with large part of the literature is that we have relaxed the commonly used assumption of homogeneous damping coefficients and considered the general case that these can be different from node to node. By using a combination of group representation theory and the solution of the quadratic eigenvalue problem, we have reduced the transient analysis of the linear swing equation in terms of a set of independent first order and second order supermodes. Each supermode is fully defined by a minimal set of parameters. This has led to a characterization of the transient error dynamics for the individual nodes of the multilayer network and the development of a method to compute the maximum value of the transient error. We have presented application of this method to two examples of interest, one of which is the IEEE 145-bus test grid, and demonstrated how it can be used to characterize the deviation between the dynamics of the full network and that of the reduced quotient network.

## Figures and Tables

**Table 1.** Initial guess calculation using a linear combination of supermodes for *η*_1_. Supermodal peak time and peak for ${\hat{\chi }}_{ij}^{\text{p}}-C_{ij}^{\left[2\right]}{\hat{f}}_{i}$ found by solving the linear ODE are in columns 2 and 3 from the left and the ones calculated using our approach are in columns 4 and 5. The table is arranged in ascending order of peak time and column 6 is the cumulative peak added in that order. The bold peak time represents the initial guess to solve (53).

*η* index	ODE Peak Time	ODE Peak	Calculated Peak Time	Calculated Peak	Cumulative Peak	Supermode Index
1	1.0359	0.0209	1.0359	0.0209	0.0209	2
	2.4354	0.0105	**2.4359**	0.0105	0.0314	1
2	1.0359	0.0021	1.0359	0.0020	0.0020	2
	2.1654	0.0377	**2.1651**	0.0377	0.0397	1

**Table 2.** Steady-state error and peak error calculation using an ODE solver in columns 2 and 3 whereas the same calculation using our approach is in columns 4 and 5. The larger of these values is the maximum shown in column 6.

*η* index	ODE Steady State	ODE Peak	Calculated Steady State	Calculated Peak	Maximum Error
1	0.0283	0.0314	0.0283	0.0314	0.0314
2	0.0300	0.0397	0.0300	0.0397	0.0397

**Table 3.** Initial guess calculation using a linear combination of supermodes for *η*_1_. Supermodal peak time and peak for ${\hat{\chi }}_{ij}^{\text{p}}-C_{ij}^{\left[2\right]}{\hat{f}}_{i}$ found by solving the linear ODE are in columns 1 and 2 from the left and the ones calculated using our approach are in columns 3 and 4. The table is arranged in ascending order of peak time and column 5 is the cumulative peak added in that order. The bold peak time represents the initial guess to solve (53).

ODE Peak Time	ODE Peak	Calculated Peak Time	Calculated Peak	Cumulative Peak	Supermode Index
0.2099	0	0.2099	0	0	3
2.0616	0.0022	2.0616	0.0022	0.0022	2
3.7260	0.0146	**3.7255**	0.0146	0.0168	1

**Table 4.** Calculation of steady-state and peak for all 3 *η*s. In columns 2 and 3 we have the steady-state error and peak error calculated using an ODE solver, whereas in columns 4 and 5 we report values calculated using our approach. Column 6 shows the maximum value between the calculated steady-state and peak errors.

*η* index	ODE Steady State	ODE Peak	Calculated Steady State	Calculated Peak	Maximum Error
1	0.0142	0.0166	0.0142	0.0166	0.0166
2	0.0406	0.0416	0.0406	0.0416	0.0416
3	0.02301	0.2742	0.2301	0.2742	0.2742
